# A National Survey of Job Satisfaction and Workload Among Emergency Medicine (EM) Residency Faculty

**DOI:** 10.7759/cureus.34982

**Published:** 2023-02-14

**Authors:** Jennifer Chapman, Martha Barrett, Meredith Thompson, Christine Stehman, Michael Hansen, Martin Wegman

**Affiliations:** 1 Emergency Medicine, HCA Florida Orange Park Hospital, Orange Park, USA; 2 Emergency Medicine, Well Span York Hospital, York, USA; 3 Emergency Medicine, University of Florida, Gainesville, USA; 4 Emergency Medicine, University of Illinois College of Medicine Peoria/OSF Healthcare, Peoria, USA; 5 Emergency Medicine, Christiana Care Health System, Newark, USA

**Keywords:** burnout, graduate medical education, emergency medicine, job satisfaction, emergency medicine faculty

## Abstract

Introduction: Faculty workload, and its relation to job satisfaction, has not been well studied in Emergency Medicine (EM).

Methods: A cross-sectional survey was conducted among EM physician faculty at 49 residency programs across the United States. We collected information on clinical and non-clinical (education, administration, and research) workload, demographics, and EM department characteristics, as well as job satisfaction measured using the Global Job Satisfaction (GJS) scale. Comparisons were made using Wilcox signed rank tests. Multivariable associations with job satisfaction were assessed in a regression model.

Results: Of 1,791 surveys sent, 265 were completed. The quantity of contracted clinical and non-clinical hours was lower than the actual clinical (difference (95% confidence interval (CI)): 2.7 (1.5-4.1)) and non-clinical hours (6.0 (3.8-8.8)) worked. Respondents preferred a distribution of 50% clinical work. However, the actual percentage was 62% (difference (95% CI): 14.4% (10.8%-17.6%)). Identifying as core faculty and required logging of non-clinical hours were associated with improved job satisfaction while increased percentage of time spent in the clinical and administrative domains were associated with significant declines.

Conclusions: The estimated actual work performed by EM physician faculty is greater than contracted and misaligned with their preferred type of work, the latter of which is associated with decreased job satisfaction. Improved job satisfaction and faculty retention might be achieved by increasing the percentage of time devoted to education and research, by increasing the core faculty contingent and by implementing tracking of non-clinical efforts.

## Introduction

An engaged, stable academic faculty serves as the foundation for emergency medicine (EM) graduate medical education. Job satisfaction, defined as the feeling of fulfillment derived from one’s work, has been closely linked to increased retention and engagement in many careers including EM [1]. While little information exists on EM faculty job satisfaction, rates of burnout, an antithetical concept, are higher in EM than other medical specialties and particularly high among academic EM faculty [2-5]. Compared to non-academic EM physicians, faculty face the additional demands of on-shift clinical instruction, increased complexity of patients treated at tertiary care teaching hospitals, didactic instruction and research [6]. Consequences of burnout include increased attrition from academics and the field, compromised quality of care, and mental illness and suicide, all of which may profoundly impact the training of the next generation of EM physicians [2,7].

It has recently been suggested that involvement in academic work such as research, teaching, and mentorship may protect against burnout and improve job satisfaction, depending on the amount of time allocated to each of these activities and its alignment with the physician’s personal preferences [8]. Yet, there is little information on academic faculty workload, and even less information on the relation of such workload to job satisfaction in EM.

Accordingly, we sought to perform a national survey of job satisfaction among EM faculty and characterize its association with demographic, departmental and workload specific factors. We were specifically interested in how actual and preferred allocation to clinical and non-clinical domains (education, research, and administration) relates to job satisfaction. Secondarily, we aimed to provide estimated work hour totals to serve as a benchmark for new and existing EM faculty and leadership and examine the concordance between actual, contracted, and preferred workload across four academic domains.

The study was reviewed and determined to be exempt from Institutional Review Board oversight at each of the investigator’s institutions. The Checklist for Reporting of Survey Studies (CROSS) was utilized to guide reporting in this manuscript and is provided in Appendix 1 [9].

## Materials and methods

Survey

A survey was developed to capture demographic and work-related characteristics of the respondents, including contracted workload, actual and preferred work-related time allocation, and job satisfaction, as measured by a validated scale [1,10]. This survey is provided is provided in Appendix 2. 

The first section of the survey aimed to capture respondents’ perceived actual hours spent per week across four major work domains (clinical, research, education, and administration) as well as their preferred work-allocation across these same domains. Examples of domain-specific activities were included with these questions. Contractual clinical and non-clinical hours were also queried. Respondents could provide these values by week, month, or year to increase accuracy, as contract structure varies widely among EM faculty. Respondents were also queried on whether they were required to account for non-clinical activities.

In the second section, we assessed job satisfaction using the Global Job Satisfaction (GJS) instrument, a 12-item scale with seven-point Likert-style response choices [1]. The GJS instrument was selected due to its succinct length, its relevance having been developed among emergency physicians (EPs), and its strong validity and reliability characteristics [10]. For example, the GJS instrument demonstrates high internal consistency (alpha = 0.91), good convergent validity with similar scales (correlation ranging from 0.50 to 0.69), and good predictive validity for EP job attrition.

The final section included 14 demographic questions. The survey was piloted among a convenience sample of academic EPs for clarity of content. First, feedback was queried among 20 academic EPs participating in the American College of Emergency Physicians’s (ACEP's) Medical Education Research Certificate workshops in 2018. Additionally, an in-person focus group of six pilot-study participants was held to assess clarity of questions and ability of questions to capture appropriate data.

Based on this feedback, questions about contract information were revised to include a branching-logic format. Additionally, several questions were revised to improve clarity and sequencing. The finalized survey encompassed 42 questions and was preceded by a brief introduction of the project (see Appendix 2 for full survey). 

Sampling

Investigators utilized a convenience sampling strategy to balance external validity and response rate, assuming that professional connections between the study investigators and leadership of programs might lead to increased survey participation. In doing so, attention was paid to geographic distribution in an attempt to improve representation. Hospital programs surveyed are listed in Appendix 3.

Investigators communicated with professional contacts via email at the targeted programs and requested assistance with distribution of the survey online through Qualtrics cloud software (Qualtrics, Provo, USA) to the academic faculty at that residency program. A general link was provided to facilitate ease of survey administration. Survey results were collected anonymously and access to results was restricted to study personnel. The sole inclusion criteria was that the respondent was an academic EM faculty member. There was no explicitly stated exclusion criteria.

Sample size was determined by balancing the consideration of survey non-response with practical constraints of the number of investigator-program connections. For this reason, power calculation was not performed. The survey was distributed in mid-spring to avoid the annual business associated with interview season and the arrival of new residents. Once the initial email was sent, the survey remained open for responses for six weeks. Reminder emails were sent two weeks after initial survey delivery and a week prior to survey closure. To estimate response rate, program contacts who assisted in distributing the survey were asked to record the number of faculty to whom they sent the survey link. If the program contact did not provide this information, the number of faculty members was determined based on counting the faculty publicly listed on the respective residency website.

Analysis

Continuous variables were summarized using medians and interquartile ranges (IQR). Categorical variables were summarized using counts and percentages. We computed the GJS summary score according to established methods. Contracted hours were standardized to a weekly basis, assuming annual and monthly hours were distributed over 52 weeks per year and 4.33 weeks per month, respectively. For sub-analyses, residency program leadership was defined as the respondent reporting their role as program director or associate/assistant program director.

Continuous outcome workload variables (workload and percentage of allocation) were found to be non-normally distributed. Thus, to examine paired differences between actual and contracted hours by domain, as well as actual and ideal percentage of allocation by domain, the non-parametric Wilcox signed-rank tests and associated 95% confidence interval (CI) were employed. Similar analyses were performed stratifying by core versus non-core faculty role.

To examine the associations between demographic and workload variables with job satisfaction, we performed multivariable step-wise backward linear regression modeling. Among all testing, a threshold p-value of 0.05 was used to determine statistical significance, with no correction for multiple testing. All responses were assumed to be unique. The small degree of missing item level data was treated as missing completely at random. No correction was performed for non-response. Weighting was incorporated into the study design and therefore no weighting was performed during analysis (Appendix 2). All analyses were performed using R Statistical software (version 4.1.2; R Core Team 2021). 

## Results

A total of 1,791 surveys were sent via email to faculty members of EM residency programs at the beginning of a six-week period from 24 February 2020 until 7 March2020. Of the 1,791 surveys sent, 265 responses were received, yielding a response rate of 14.8%. Reasons for non-participation are unknown. Demographic and academic characteristics of the respondents are shown in Table 1. Of note, the respondents were predominantly male (66%), married (80%), white (75.6%), and had at least one dependent under the age of 18 living in their household (59.6%) The median age was 40 (IQR; 35, 48) and the median number of years of post-graduate academic EM experience was 9.3 years (IQR; 3, 13). There was a predominance of university setting (56%) and 23% were program directors or assistant program directors. Respondents were distributed across the US, and response rates were similar across regions. 

**Table 1:** Respondent characteristics Numbers are N (%) unless otherwise noted. Q1 = 25th percentile, Q3 = 75th percentile.

Decline to answer	5 (1.9%)
Female	84 (31.7%)
Male	176 (66.4%)
Age (years)	
Median (Q1, Q3)	42 (35, 48)
Missing	1 (0.4%)
Race	
American Indian or Alaskan Native	1 (0.4%)
Asian	28 (10.6%)
Black or African American	8 (3.0%)
Hispanic or Latinx	6 (2.3%)
Prefer not to answer	14 (5.3%)
Two or more races	7 (2.6%)
White (not of Hispanic origin)	200 (75.5%)
Missing	1 (0.4%)
Relationship status	
Divorced	10 (3.8%)
Domestic partner	8 (3.0%)
Married	212 (80.0%)
Never married	1 (0.4%)
Other	2 (0.8%)
Prefer not to answer	4 (1.5%)
Single	27 (10.2%)
Widowed	1 (0.4%)
Dependents < 18 years old	
0	107 (40.4%)
1	38 (14.3%)
2	87 (32.8%)
3+	33 (12.5%)
Dependents ≥ 18 years old	
0	224 (84.5%)
1	30 (11.3%)
2	6 (2.3%)
3+	5 (1.9%)
Time after training (years)	
Median (Q1, Q3)	12 (5.0, 17)
Time after training in academics (years)	
Median (Q1, Q3)	9.3 (3.0, 13)
Full or part-time	
Full-time	253 (95.5%)
Part-time	10 (3.8%)
Missing	2 (0.8%)
Core faculty	
No	52 (19.6%)
Yes	206 (77.7%)
Missing	7 (2.6%)
Program director/assistant program director	
No	200 (75.5%)
Yes	60 (22.6%)
Missing	5 (1.9%)
Hospital type	
Community	46 (17.4%)
County or city	48 (18.1%)
Large-tertiary care (not university affiliated)	21 (7.9%)
Other	3 (1.1%)
University	147 (55.5%)
Geographical region	
Central East (IN, KY, MI, OH, TN)	5 (1.9%)
Mid Atlantic (DC, DE, MD, NC, NJ, PA, VA, WV)	89 (33.6%)
North Central (AR, IA, IL, KS, MN, MO, ND, NE, OK, SD, WI)	22 (8.3%)
Northeast (CT, MA, ME, NH, NY, RI, VT)	48 (18.1%)
Southeast (Puerto Rico, AL, FL, GA, LA, MS, SC)	42 (15.8%)
Southwest (AZ, CO, NM, NV, TX, UT)	44 (16.6%)
West (CA, ID, MT, OR, WA, WY)	15 (5.7%)

Further description of geographic composition of programs and survey responses is shown in Table 2. 

**Table 2:** Geographic data

	Composition of Programs Nationally in 2020	Composition of our Survey Sample	Composition of Survey Responses
Northeast (CT, MA, ME, NH, NY, RI, VT)	17.27%	12.24%	18.1%
Mid Atlantic (DC, DE, MD, NC, NJ, PA, VA, WV)	18.88%	36.73%	33.6%
Southeast (PR, AL, FL, GA, LA, MS, SC)	14.86%	18.37%	15.8%
Central East (IN, KY, MI, OH, TN)	20.08%	4.08%	1.9%
North Central (AR, IA, IL, KS, MS, MO, ND, NE, OK, SD, WI)	12.45%	10.20%	8.3%
Southwest (AZ, CO, NV, TX, UT)	7.63%	10.20%	16.6%
West (CA, ID, MT, OR, WA, WY, AS)	8.84%	8.16%	5.7%

The median number of estimated actual total hours worked per week was 43 (IQR; 20, 25), with 24 (20,25) allocated to clinical activities and 16 (10, 20) allocated to non-clinical activities. (Unlike arithmetic averages, the sum of the medians for individual domains will not necessarily equal the overall median.) Among respondents, 80% knew their contracted number of clinical hours, 14% were able to estimate a value and 5.3% reported not knowing their contracted number of clinical hours. As shown in Table 3, respondents reported working 2.7 more clinical hours (1.5, 4.1) and six more non-clinical hours (3.8, 8.8) per week than their respective contracted hours for these domains. This non-contracted work reflects 11% and 37.5% of all clinical and non-clinical time, respectively. Respondents preferred to spend a median of 50% of their work-related time on clinical activities, however the actual percentage was 62% (Wilcox signed rank difference, (95% CI): 14.4% (10.8%, 17.6%)). Respondents also reported lower time allocation to education and research than desired: (-11.4% (-13.3%, -9.7%)) and (-2% (-3.4%, -0.2%)), respectively. There was no statistical difference between actual and desired administrative work allocation.

**Table 3:** Actual, contracted, and preferred workload by domain Median (Q1, Q3) hours per week are shown. Q1 = 25th percentile, Q3 = 75th percentile. Δ (95% CI) represents paired Wilcox signed rank differences and associated 95% CI. CI: Confidence interval.

Work domain	Hours per week			% Allocation		
	Actual	Contracted	Δ (95% CI)	Actual	Preferred	Δ (95% CI)
Clinical	24 (20,25)	22 (16,22)	2.7 (1.5,4.1)	62 (41,59)	50 (32,45)	14.4 (10.8,17.6)
Non-Clinical	16 (10,20)	12 (5,13)	6 (3.8,8.8)	38 (25,41)	50 (42,55)	-14.4 (-17.6,-10.8)
Education	5 (4,7)			13 (8,15)	25 (19,26)	-11.4 (-13.3,-9.7)
Administration	5 (2,9)			12 (5,19)	11 (5,17)	0 (-1.3,2.5)
Research	2 (0,4)			4 (0,7)	7 (0,11)	-2 (-3.4,-0.2)
Total	43 (38,45)	36 (30,35)	9 (6.5,11)			

77% of respondents identified as core faculty and 23% reported they were program directors or associate/assistant program directors for an EM residency program. An ad-hoc analysis was performed to examine differences in actual and contracted work hours and percentage allocation to work domains between non-residency leadership core and non-core faculty (see Table 4 and 5). Core and non-core faculty reported similar contracted hours. However, non-core faculty reported working more clinical hours and fewer non-clinical hours, with no statistically significant difference in total hours worked. There was no significant difference in desired workload allocation between core and non-core faculty respondents. Estimated actual, contracted and preferred workload by domain was also examined for residency program leadership (Table 6). Findings were very similar to the aggregate sample, except that there was preference for a greater portion of non-clinical time across all domains.

**Table 4:** Work hours by domain for core and non-core faculty, excluding residency program leadership Median (Q1, Q3) hours per week are shown. Q1 = 25th percentile, Q3 = 75th percentile. Δ (95% CI) represents paired Wilcox signed rank differences and associated 95% CI. CI: Confidence interval.

Work domain	Actual hours per week			Contracted hours per week		
	Core	Non-Core	Δ (95% CI)	Core	Non-Core	Δ (95% CI)
Clinical	24 (20,24)	29 (24,29)	-4 (-7,-1)	23 (17,22)	22 (15,21)	0 (-2.2,2.3)
Non-Clinical	18 (10,21)	11 (6,13)	7 (3,10)	12 (5,13)	7 (4,8)	4 (-0.4,8.2)
Education	5 (4,7)	4 (2,5)	1 (0,2)			
Administration	5 (2,9)	4 (1,6)	2 (0,3.5)			
Research	2 (0,5)	1 (0,2)	1 (0,1)			
Total	44 (39,45)	40 (35,42)	3 (-1,6)	33 (29,31)	36 (30,35)	1.8 (-1.5,7.5)

**Table 5:** Percent work allocation by domain for core and non-core faculty, excluding residency program leadership Median (Q1, Q3) hours per week are shown. Q1 = 25th percentile, Q3 = 75th percentile. Δ (95% CI) represents paired Wilcox signed rank differences and associated 95% CI. CI: Confidence interval.

Work domain	Actual % allocation			Preferred % allocation		
	Core	Non-Core	Δ (95% CI)	Core	Non-Core	Δ (95% CI)
Clinical	61 (40,57)	74 (60,71)	-13.5 (-20.5,-6.8)	48 (32,45)	50 (40,52)	-9 (-12,0)
Non-clinical	39 (28,43)	26 (15,29)	13.5 (6.8,20.5)	52 (46,55)	50 (39,48)	9 (0,12)
Education	12 (8,15)	9 (5,12)	3.2 (0.4,5.7)	25 (16,25)	23 (14,23)	1 (-2,6)
Administration	12 (5,19)	9 (3,13)	3.7 (0.3,7.4)	10 (5,17)	10 (5,15)	0 (-1,5)
Research	4 (0,9)	3 (0,5)	1.3 (0,2.9)	7 (0,13)	6 (0,9)	0 (0,5)

**Table 6:** Actual, contracted, and preferred workload by domain for residency program leadership Median (Q1, Q3) hours per week are shown. Q1 = 25th percentile, Q3 = 75th percentile. Δ (95% CI) represents paired Wilcox signed rank differences and associated 95% CI. CI: Confidence interval.

Work domain	Hours per week			% Allocation		
	Actual	Contracted	Δ (95% CI)	Actual	Preferred	Δ (95% CI)
Clinical	23 (18,23)	21 (15,21)	1.5 (-0.5,4)	48 (39,50)	40 (30,40)	10 (3.3,16.1)
Non-clinical	22 (14,24)	15 (12,16)	7 (1.6,12.3)	52 (38,50)	60 (50,60)	-10 (-16.1,-3.3)
Education	8 (6,9)			19 (14,20)	30 (25,32)	-11 (-14.1,-7.6)
Administration	9 (5,12)			20 (11,24)	17 (10,18)	4.9 (0,10)
Research	2 (1,3)			5 (2,6)	10 (3,10)	-2.7 (-5,-0.5)
Total	44 (40,47)	36 (33,36)	9.5 (6,13.7)			

The median GJS score among respondents in our survey was 12 (IQR: 2, 22), with 22% of faculty reporting GJS scores 0 or lower. For context, GJS has a theoretical range of -36 to 36, with increasing scores associated with improved job satisfaction. Prior predictive validity research found an average GJS of 11.8 among EM faculty retaining their positions and an average value of 1.8 among faculty leaving the field of EM. Regression analysis (Table 7) revealed that job satisfaction was independently associated with several work characteristics. For every 10% increase in clinical allocation of workload, average GJS decreased by 3.5 points. For every 10% increase in time spent in the administrative domain, GJS, on average, decreased by 2.6 points. This means that increased percentage allocation to clinical and administrative work (and thus decreased allocation to education and research related work) was associated with decreased job satisfaction after controlling for all other studied factors. Conversely, core faculty status was associated with a seven-point increase in GJS, on average, compared with non-core faculty status. Requirement of logging of non-clinical work activities was associated with a 3.7-point increase in GJS. For every 10% increase in the ideal percentage of clinical work, GJS increased by 2.6 points, indicating that faculty who prefer to work more clinical hours had greater job satisfaction after accounting for other study variables.

**Table 7:** Multivariable associations with job satisfaction * Indicates significance at the 0.05 level. CI: Confidence interval.

Variable	Estimate (95% CI)
Relationship status (other)	-3.1 (-11, 4.5)
Relationship status (single)	-5.7 (-11, 0.042)
Core faculty	6.9 (2.4, 11)*
Required non-clinical logging	3.7 (0.16, 7.2)*
Actual % allocation (clinical)	-0.35 (-0.52, -0.18)*
Actual % allocation (administrative)	-0.26 (-0.47, -0.059)*
Preferred % allocation (clinical)	0.26 (0.11, 0.41)*
Preferred % allocation (administrative)	0.18 (-0.021, 0.38)
Total hours worked	-0.18 (-0.36, 0.0077)

## Discussion

To our knowledge, this is the first national survey of EM faculty examining workload, relative work domain allocation, and the relationship between these factors and job satisfaction. Such information can serve as a guide for new and existing faculty and institutional leadership on work hours and work allocation, both in terms of benchmarking against current practices as well as in efforts to improve faculty job satisfaction and reduce attrition. Additionally, although we did not directly assess burnout, these findings could be interpreted for use in workplace initiatives to reduce burnout and improve well-being of the EM workforce.

Our study found that core and non-core faculty report working more hours, both clinically and non-clinically, than is stated in their contracts. Assuming 46 working weeks per year, this equates to the typical respondent working approximately 124 more clinical and 276 more nonclinical hours annually than contracted. Based on this, accounting of academic workload should include non-contracted work. If not, total work effort will be significantly underestimated, with more than twofold greater absolute and more than 3.5-fold greater relative underestimation of non-clinical efforts. Even so, this difference between estimated actual and contracted work was not independently associated with job satisfaction in our model. Further research will need to investigate whether this non-contracted time is compensated and confirm the relationship between this extra work effort and job satisfaction.

Our survey did not distinguish between administrative, educational, and research effort dedicated to the faculty’s residency program or other purposes such as departmental operations or undergraduate medical education. Even so, the typical core faculty member reported spending 18 hours per week on non-clinical activities, seven of which were devoted to education and research-related work alone. These findings are particularly salient given the recent alterations to EM residency program core faculty hour standards proposed by the Accreditation Council for Graduate Medical Education (ACGME) [11]. The new proposed minimum threshold for non-clinical activity is 10% full-time equivalent (FTE), which amounts to to approximately four hours per week based on the median hours worked per week in our sample. This is less than 25% of the estimated median actual time and one-third the median contracted time currently devoted to non-clinical efforts by core faculty in our survey.

We were surprised that 5% of academic EM physicians were unaware of the clinical hours they are contracted to work. Why academic EM physicians are unaware of contracted clinical hours and whether this number is consistent in community EM practice should be a topic of further research.

We found that faculty spend more time performing administrative and clinical work and less time on education and research than preferred. In our model, this discordance was linked to decreased job satisfaction, with a 10% absolute change in either measure equivalent in magnitude to approximately one-third the score difference between faculty who remained in their positions and those who left the EM workforce entirely. Preference for increased allocation to clinical work appeared to be protective, as it was associated with increased job satisfaction in our model. Alarmingly, 22% of respondents had GJS scores 0 or lower, a threshold associated with a positive predictive value of 22% for attrition from the field. Stated another way, more than one in five of these faculty could be expected to leave EM.

Serving as a core faculty member demonstrated one of the strongest associations with job satisfaction, independent of the increased allocation to non-clinical work observed by this group. Future research will be needed to further understand the factors underlying this, such as engagement in more satisfying domain-specific work, feeling more valued for their work, increased reputational or financial gains owing from this role, or more innate differences in job satisfaction. Additionally, the requirement of logging non-clinical activity was also associated with increased job satisfaction. Reasons for this finding are not entirely clear but could be explained by the fact that such logging may be required when non-clinical hours are more closely remunerated. For example, a study of departed faculty from a single medical school observed higher rates of attrition among academic physicians who perceive lack of compensation for their academic work [12]. Alternatively, this could signal that leadership is taking a more active role in ensuring faculty’s efforts are aligned with departmental priorities, and that faculty are engaged in work which is more valued and recognized by the department.

Limitations

Our study has several limitations. First, the estimated response rate was only 14.8%. Low response rates can impact survey data in two ways: by decreasing precision (which subsequently increases the chance of a Type I error) and by decreasing generalizability, particularly if there was differential non-response. Given that we were able to identify several statistically significant findings and that confidence intervals were reasonable in size, our precision seemed adequate. With regards to assessing generalizability of our respondent pool, there is limited data available on the demographic characteristics of the academic EM workforce against which to compare. As a rough approximation, the American Association of Medical Colleges (AAMC) publishes specialty demographic statistics based on the American Medical Association’s (AMA's) Physician Master File. In the most recent data from 2018-2019, 71.7% of the entire EM physician workforce was male and 70% were white, mirroring our respondent gender and racial breakdown [13-15]. However, our sample appears younger (89% vs 65.1% aged less than 55 years) than the field as a whole. 

Furthermore, our sample was limited to those programs with whom investigators had contacts, thus representing a convenience sample which may further impact generalizability. Efforts were made to ensure a good degree of geographic balance. However, the Mid-Atlantic and Southwest were overrepresented and the Central East was substantially under-represented (see Appendix 3). The impact of this imbalance is dependent on the degree of interregional variation on the studied characteristics.

In an attempt to improve survey convenience and response rate, a general survey link was emailed to each program contact to distribute to individuals they considered “academic faculty” at their institution. Therefore, multiple participation and participation of individuals that might not typically be considered academic faculty was possible. Additionally, actual and contracted hours were self-reported and thus subject to error. Differences in contract structure amongst employers and independent contractors may have rendered some of the questions unanswerable.

Lastly, survey distribution occurred during the start of the global pandemic of coronavirus disease 2019 (24 February 2020 to 7 March 2020). It is unclear how this may have impacted the response rate as well as estimations of workload and job satisfaction.

## Conclusions

Based on this national survey, the estimated actual work performed by EM physician faculty is greater than contracted and misaligned with their preferred type of work. Additionally, a substantial portion of faculty are at risk for attrition. There is potential to retain faculty and improve job satisfaction by reallocating work-related activities, specifically by reducing clinical hours, increasing the percentage of non-clinical time devoted to education and research endeavors, and having a system to track non-clinical work. 

## Appendices

Appendix 1

**Table 8:** Checklist for Reporting Of Survey Studies (CROSS)

Section/topic	Item	Item description	Reported on page #
Title and abstract			
Title and abstract	1a	State the word “survey” along with a commonly used term in title or abstract to introduce the study’s design.	
	1b	Provide an informative summary in the abstract, covering background, objectives, methods, findings/results, interpretation/discussion, and conclusions.	
Introduction			
Background	2	Provide a background about the rationale of study, what has been previously done, and why this survey is needed.	
Purpose/aim	3	Identify specific purposes, aims, goals, or objectives of the study.	
Methods			
Study design	4	Specify the study design in the methods section with a commonly used term (e.g., cross-sectional or longitudinal).	
	5a	Describe the questionnaire (e.g., number of sections, number of questions, number and names of instruments used).	
Data collection methods	5b	Describe all questionnaire instruments that were used in the survey to measure particular concepts. Report target population, reported validity and reliability information, scoring/classification procedure, and reference links (if any).	
	5c	Provide information on pretesting of the questionnaire, if performed (in the article or in an online supplement). Report the method of pretesting, number of times questionnaire was pre-tested, number and demographics of participants used for pretesting, and the level of similarity of demographics between pre-testing participants and sample population.	
	5d	Questionnaire if possible, should be fully provided (in the article, or as appendices or as an online supplement).	
Sample characteristics	6a	Describe the study population (i.e., background, locations, eligibility criteria for participant inclusion in survey, exclusion criteria).	
	6b	Describe the sampling techniques used (e.g., single stage or multistage sampling, simple random sampling, stratified sampling, cluster sampling, convenience sampling). Specify the locations of sample participants whenever clustered sampling was applied.	
	6c	Provide information on sample size, along with details of sample size calculation.	
	6d	Describe how representative the sample is of the study population (or target population if possible), particularly for population-based surveys.	
Survey administration	7a	Provide information on modes of questionnaire administration, including the type and number of contacts, the location where the survey was conducted (e.g., outpatient room or by use of online tools, such as SurveyMonkey).	
	7b	Provide information of survey’s time frame, such as periods of recruitment, exposure, and follow-up days.	
	7c	Provide information on the entry process: –>For non-web-based surveys, provide approaches to minimize human error in data entry. –>For web-based surveys, provide approaches to prevent “multiple participation” of participants.	
Study preparation	8	Describe any preparation process before conducting the survey (e.g., interviewers’ training process, advertising the survey).	
Ethical considerations	9a	Provide information on ethical approval for the survey if obtained, including informed consent, institutional review board [IRB] approval, Helsinki declaration, and good clinical practice [GCP] declaration (as appropriate).	
	9b	Provide information about survey anonymity and confidentiality and describe what mechanisms were used to protect unauthorized access.	
Statistical analysis	10a	Describe statistical methods and analytical approach. Report the statistical software that was used for data analysis.	
	10b	Report any modification of variables used in the analysis, along with reference (if available).	
	10c	Report details about how missing data was handled. Include rate of missing items, missing data mechanism (i.e., missing completely at random [MCAR], missing at random [MAR] or missing not at random [MNAR]) and methods used to deal with missing data (e.g., multiple imputation).	
	10d	State how non-response error was addressed.	
	10e	For longitudinal surveys, state how loss to follow-up was addressed.	
	10f	Indicate whether any methods such as weighting of items or propensity scores have been used to adjust for non-representativeness of the sample.	
	10g	Describe any sensitivity analysis conducted.	
Results			
Respondent characteristics	11a	Report numbers of individuals at each stage of the study. Consider using a flow diagram, if possible.	
	11b	Provide reasons for non-participation at each stage, if possible.	
	11c	Report response rate, present the definition of response rate or the formula used to calculate response rate.	
	11d	Provide information to define how unique visitors are determined. Report number of unique visitors along with relevant proportions (e.g., view proportion, participation proportion, completion proportion).	
Descriptive results	12	Provide characteristics of study participants, as well as information on potential confounders and assessed outcomes.	
Main findings	13a	Give unadjusted estimates and, if applicable, confounder-adjusted estimates along with 95% confidence intervals and p-values.	
	13b	For multivariable analysis, provide information on the model building process, model fit statistics, and model assumptions (as appropriate).	
	13c	Provide details about any sensitivity analysis performed. If there are considerable amount of missing data, report sensitivity analyses comparing the results of complete cases with that of the imputed dataset (if possible).	
Discussion			
Limitations	14	Discuss the limitations of the study, considering sources of potential biases and imprecisions, such as non-representativeness of sample, study design, important uncontrolled confounders.	
Interpretations	15	Give a cautious overall interpretation of results, based on potential biases and imprecisions and suggest areas for future research.	
Generalizability	16	Discuss the external validity of the results.	
Other sections			
Role of funding source	17	State whether any funding organization has had any roles in the survey’s design, implementation, and analysis.	
Conflict of interest	18	Declare any potential conflict of interest.	
Acknowledgements	19	Provide names of organizations/persons that are acknowledged along with their contribution to the research.	

Appendix 2

Dear colleagues,

We are a multi-institutional work group studying workload, compensation, and job satisfaction. 

This survey takes approximately 5 minutes to complete and collects information about perceived work hours, allocation of hours, as well as job satisfaction. We are looking for responses from all physician faculty currently working at ACGME-approved US emergency medicine residency teaching sites.     

This is an anonymous survey used for research. Data will be viewed by our workgroup and will not include personal identifiers. Whether you decide to participate in this research is entirely your choice. You can decide not to participate or to halt participation at any time for any reason by contacting the principal investigator, Michael Hansen, MD by email (see below). Doing so will not affect how you are treated at your institution and will not affect your educational standing or employment.     

We hope that you will decide to participate in this short survey. The results of this study may be useful in shaping departmental policies to maximize overall faculty physician wellness and retention.     

The survey results will be used for research purposes and eventually shared. It has been declared exempt by the Institutional Review Board at each participating institution. Investigators for this study are listed below and can be contacted for additional information.     

We thank you for your assistance.    

Start of Block: Block 1

RSTU Q4 The tasks of an academic clinician have been broken down into four domains in the literature: patient care, education, administration, and research. Please estimate on average the actual number of hours you spend on each type of task weekly.

o R Patient Care  Examples: clinical hours, on-shift teaching, charting  (1) ________________________________________________

o S Education  Examples: lectures, labs, simulation, resident conference attendance, mentoring  (2) ________________________________________________

o T Administration  Examples: quality improvement, scheduling, residency committees, chair of department  (3) ________________________________________________

o U Research  Examples: IRB participation, research hours, editorial board membership  (4) ________________________________________________

VWXY Q6 In your ideal job, how would you prefer the breakdown of each domain (adding up to 100 percent)?

 _______ V Patient Care (1)

 _______ W Education (2)

 _______ X Administration (3)

 _______ Y Research (4)

Z Q34 Do you know how many clinical hours you are contracted to work?

o Yes I know the number in my contract  (1)

o I know there is a number and I can make an estimate  (2)

o I have no idea  (4)

Skip To: Q20 If Do you know how many clinical hours you are contracted to work? = Yes I know the number in my contract

Skip To: Q20 If Do you know how many clinical hours you are contracted to work? = I know there is a number and I can make an estimate

Skip To: Q34 If Do you know how many clinical hours you are contracted to work? = I have no idea

AA Q20 Does your contract specify your contracted clinical (patient care) hours as weekly, monthly, or yearly hours?

▼ Weekly (1) ... Yearly (3)

Display This Question:

If Does your contract specify your contracted clinical (patient care) hours as weekly, monthly, or y... = Weekly

AB Q12 How many hours per week are specified?

_______________________________________________________________

Display This Question:

If Does your contract specify your contracted clinical (patient care) hours as weekly, monthly, or y... = Monthly

AC Q13 How many hours per month are specified?

________________________________________________________________

Display This Question:

If Does your contract specify your contracted clinical (patient care) hours as weekly, monthly, or y... = Yearly

AD Q14 How many hours per year are specified?

________________________________________________________________

AE Q34 Please choose which you think most applies to your perception of the number of hours you are contracted to work clinically. 

o I am working less than in my contract  (1)

o I am working more than in my contract  (2)

o I am working what is specified in my contract  (3)

AF Q15 Does your contract specify your contracted non-clinical (education, administrtion, research) hours as weekly, monthly, or yearly hours?

▼ Weekly (1) ... Not Specified (4)

Display This Question:

If Does your contract specify your contracted non-clinical (education, administrtion, research) hour... = Weekly

AG Q16 How many hours per week are specified?

________________________________________________________________

Display This Question:

If Does your contract specify your contracted non-clinical (education, administrtion, research) hour... = Monthly

AH Q17 How many hours per month are specified?

________________________________________________________________

Display This Question:

If Does your contract specify your contracted non-clinical (education, administrtion, research) hour... = Yearly

AI Q18 How many hours per year are specified?

________________________________________________________________

AJ Q58 Please choose which you think most applies to you perception of the number of hours you are contracted to work outside of your clinical duties.

o I am working less than in my contract  (1)

o I am working more than in my contract  (2)

o I am working what is specified in my contract  (3)

o My contract does not specify  (4)

AK Q35 Do you have to account for your non-clinical (education, administration, research) time in any formal way?

▼ Yes (1) ... No (2)

AL Q19 Are you compensated separately for administrative tasks?

▼ Yes (23) ... No (24)

End of Block: Block 1

Start of Block: GJS

AM Q45 I feel stagnant in my present position. 

o -3     (Strongly Disagree)  (1)

o -2  (2)

o -1  (3)

o 0     (Neutral)  (4)

o 1  (5)

o 2  (6)

o 3     (Strongly Agree)  (7)

AN Q47 I am fed up with my job and would like to work in another hospital. 

o -3 (Strongly Disagree)  (1)

o -2  (2)

o -1  (3)

o 0 (Neutral)  (4)

o 1  (5)

o 2  (6)

o 3 (Strongly Agree)  (7)

AO Q48 I feel my current position is right for me. 

o -3 (Strongly Disagree)  (1)

o -2  (2)

o -1  (3)

o 0 (Neutral)  (4)

o 1  (5)

o 2  (6)

o 3 (Strongly Agree)  (7)

AP Q49 I feel I am achieving worthwhile results through my work. 

o -3 (Strongly Disagree)  (1)

o -2  (2)

o -1  (3)

o 0 (Neutral)  (4)

o 1  (5)

o 2  (6)

o 3 (Strongly Agree)  (7)

AQ Q50 I feel like I am at the end of my rope. 

o -3 (Strongly Disagree)  (1)

o -2  (2)

o -1  (3)

o 0 (Neutral)  (4)

o 1  (5)

o 2  (6)

o 3 (Strongly Agree)  (7)

AR Q51 I feel I am becoming burnt out from my work. 

o -3 (Strongly Disagree)  (1)

o -2  (2)

o -1  (3)

o 0 (Neutral)  (4)

o 1  (5)

o 2  (6)

o 3 (Strongly Agree)  (7)

AS Q52 I feel enthusiastic about my work. 

o -3 (Strongly Disagree)  (1)

o -2  (2)

o -1  (3)

o 0 (Neutral)  (4)

o 1  (5)

o 2  (6)

o 3 (Strongly Agree)  (7)

AT Q53 After working in this department, crisis seems repetitive. 

o -3 (Strongly Disagree)  (1)

o -2  (2)

o -1  (3)

o 0 (Neutral)  (4)

o 1  (5)

o 2  (6)

o 3 (Strongly Agree)  (7)

AU Q54 I am pleased with what I am accomplishing in life. 

o -3 (Strongly Disagree)  (1)

o -2  (2)

o -1  (3)

o 0 (Neutral)  (4)

o 1  (5)

o 2  (6)

o 3 (Strongly Agree)  (7)

AV Q55 I feel frustrated by my job. 

o -3 (Strongly Disagree)  (1)

o -2  (2)

o -1  (3)

o 0 (Neutral)  (4)

o 1  (5)

o 2  (6)

o 3 (Strongly Agree)  (7)

AW Q56 I feel enthusiastic about my present position at work. 

o -3 (Strongly Disagree)  (1)

o -2  (2)

o -1  (3)

o 0 (Neutral)  (4)

o 1  (5)

o 2  (6)

o 3 (Strongly Agree)  (7)

AX Q57 I would like to change to another type of practice. 

o -3 (Strongly Disagree)  (1)

o -2  (2)

o -1  (3)

o 0 (Neutral)  (4)

o 1  (5)

o 2  (6)

o 3 (Strongly Agree)  (7)

End of Block: GJS

Start of Block: Block 3

AY Q23 What is your age?

________________________________________________________________

AZ Q24 What is your gender?

▼ Male (1) ... Prefer to self describe (6)

Skip To: Q44 If What is your gender? = Prefer to self describe

Skip To: Q25 If What is your gender? = Female

Skip To: Q25 If What is your gender? = Decline to answer

Skip To: Q25 If What is your gender? = Male

Skip To: Q25 If What is your gender? = Other

BA Q44 Please describe your gender

________________________________________________________________

BB Q25 What is your race?

▼ White (not of Hispanic origin) (1) ... Prefer not to answer (8)

BC Q26 Which relationship status best describes you?

▼ Single (1) ... Prefer not to answer (7)

BDBE Q27 How many dependents do you have at home?

o BD Less than 18 years  (1) ________________________________________________

o BE 18 years or older  (2) ________________________________________________

BF Q28 How many years have you been a practicing emergency physician post-training?

________________________________________________________________

BG Q29 How many years have you been a practicing academic emergency physician post-residency?

________________________________________________________________

BH Q8 Do you work full or part-time?

▼ Full-time (1) ... Part-time (2)

BI Q9 Are you considered core faculty by ACGME for your residency?

▼ Yes (17) ... No (18)

BJ Q10 Are you a program director or associate/assistant program director

▼ Yes (23) ... No (24)

BK Q30 What type of hospital do you primarily practice in?

▼ University (1) ... Other (6)

Display This Question:

If What type of hospital do you primarily practice in? = Other

BL Q31 If you chose other, how would you describe your setting?

________________________________________________________________

BM Q32 What region do you practice in?

▼ Northeast (CT, MA, ME, NH, NY, RI, VT) (1) ... West (CA, ID, MT, OR, WA, WY) (7)

End of Block: Block 3

Appendix 3

List of Academic Institutions for Survey Release (numbers and names based on ERAS)

Reviewed in 2018 at time of survey development.

Overall Programs: 249 Total (19.6%) #49

Overall Academic Faculty: 1,791

Northeast (CT-2, MA-5, ME-1, NH-1, NY-31, RI-2, VT-1) 

UMMS-Baystate (MA)  - 43

University of Massachusetts (MA) - 133

New York Presbyterian Hospital (Columbia University) (NY) - 90

Orange Regional Medical Center (NY) - 6

SUNY Health Science Center at Brooklyn (NY) - 55

Zucker School of Medicine at Hofstra/Northwell at Staten Island University Hospital (NY) - 55

Mid Atlantic (DC-2, DE-1, MD-2, NC-7, NJ-9, PA-20, VA-5, WV-2) 

MedStar Health/ Georgetown-Washington Hospital Center (DC) - 12

Christiana Care Health Services (DE) - 20

Carolinas Medical Center (NC)  - 40

Wake Forest University School of Medicine (NC) - 42

Cooper Medical School of Rowan University/Cooper University Hospital (NJ) - 30

Inspira Health Network Program (NJ) - 14

Rutgers Robert Wood Johnson Medical School (NJ) - 22

St Joseph’s University Medical Center (NJ) -13

Albert Einstein Healthcare Network (PA) - 50

Crozer-Chester Medical Center (PA) - 10

Geisinger Health System (PA) - 20

Lehigh Valley Health Network/University of South Florida College of Medicine (PA) - 20

Penn State Milton S Hershey Medical Center (PA) - 30

Sidney Kimmel Medical College at Thomas Jefferson University Hospital (PA) - 40

Temple University Hospital (PA) - 53

University of Pennsylvania Health System (PA) - 62

York Hospital (PA) - 40

University of Virginia Medical Center (VA) - 33

Southeast (PR-2, AL-2, FL-19, GA-4, LA-4, MS-2, SC-4) 

University of Alabama Medical Center (AL) - 30

University of Central Florida/HCA GME Consortium (Ocala) (FL) - 7

Palm Beach Consortium for GME (FL) (St. Lucie) - 6

Orange Park Medical Center (FL) - 11

University of Florida (FL) (Gainesville) - 56

University of Florida College of Medicine Jacksonville (FL) -  50

Emory University School of Medicine (GA) - 100

Coliseum Medical Centers (GA) - 5

Grand Strand Medical Center (SC) - 14

Central East (IN-1, KY-2, MI-25, OH-18, TN-4)

University of Kentucky (KY) - 32

OhioHealth/Doctors Hospital (OH) - 17

North Central (AR-2, IA-1, IL-11, KS-1, MN-3, MO-5, ND-0, NE-1, OK-5, SD-0, WI-2) 

Cook County Health and Hospitals System (IL) - 32

McGaw Medical Center of Northwestern University (IL) - 30

Loyola University Medical Center (IL) - 35

University of Illinois College of Medical at Peoria (IL) - 13

Hennepin Healthcare (MN) - 35

Southwest (AZ-4, CO-1, NV-3, TX-10, UT-1)

Baylor College of Medicine (TX) - 30 

University of Texas Health Science Center San Antonio Joe and Teresa Lozano Long School of Medicine  (TX) - 44

University of Texas Health Science Center at Houston (TX) - 35

University of Texas Southwestern Medical Center (TX) - 100

Texas Tech University HSC El Paso (TX) - 19

West (CA-19, ID-0, MT-0, OR-1, WA-1, WY-0) 

Loma Linda University Health Education Consortium (CA) - 67

Riverside Community Hospital - UC Riverside (CA) - 15

Los Angeles County-Harbor-UCLA Medical Center (CA) - 34

University of Oregon (OR) - 41
